# Supplementary material for: Identifying the impact of social influences in health-related discrete choice experiments
Source: PLoS One. 2022 Oct 19;17(10):e0276141. doi: 10.1371/journal.pone.0276141 (PMC9581381; doi:10.1371/journal.pone.0276141)
Supplement: S1 Appendix — (DOCX) [file pone.0276141.s001.docx]

**S1_Appendix: Discrete Choice Experiment Design**

We generated a DCE design consisting of 600 choice tasks blocked into 50 sub-designs using NGene software (2012). This DCE design was optimal to estimate a multinomial logit (MNL) model, based on a main-effects utility function. The prior preference information (attribute weights) as required for the Bayesian efficient optimization approach was obtained from best guess priors using expert judgement. Each respondent was randomly assigned to a sub-design containing 12 discrete choice tasks each.

To reduce respondent burden further and to be as clear as possible, the attribute levels of ‘the delay or split scheduled vaccination’ profile were presented as a direct comparison with the ‘fixed recommended vaccination schedule’ profile. To illustrate, opting for the ‘recommended vaccination schedule’ profile meant always a total number of nine injections. If the total number of injections was 12 for ‘the delay or split scheduled vaccination’ profile, we presented these 12 injections in the DCE choice task as ‘3 more injections than the recommended schedule’. The ‘opt out’ (i.e., the ‘no vaccination’ profile) alternative was necessary in each choice task to mimic real life childhood vaccination decisions as childhood vaccination is not mandatory.

*References*

ChoiceMetrics. Ngene 1.1.1 User Manual & Reference Guide, Australia. (2012).
