# Supplementary material for: Identifying the impact of social influences in health-related discrete choice experiments
Source: PLoS One. 2022 Oct 19;17(10):e0276141. doi: 10.1371/journal.pone.0276141 (PMC9581381; doi:10.1371/journal.pone.0276141)
Supplement: S2 Appendix — (DOCX) [file pone.0276141.s002.docx]

**S2_Appendix: Sample Size Calculation**

For this study, the formal parametric sample size calculation approach for DCE studies from de Bekker-Grob et al (2015) (i.e. taking the DCE design and priors/final estimates into account) cannot be used as that approach is for unlabelled DCE studies and MNL models only. However, the general sample-size method given in Louviere, Hensher and Swait (2000) can be used to provide a sample size estimate that does not take the priors into account, and so is far more conservative than would be the case otherwise. But nonetheless, it is still useful as a comparison point to the sample size that was actually collected in this study (N=604). Consider that the aim is to try to estimate a (critical) multinomial probability of around *p*≈0.20, which is approximately the proportion of choices actually observed for the designed alternative in the DCE. The appropriate formula from Louviere, Hensher and Swait (2000) is expression (9.5):

$$n\geq\frac{q}{rpa^{2}}\left[\Phi^{-1}\left(\frac{1+\alpha}{2}\right)\right]^{2}$$

where *n* is the required minimum sample size, *p* has been defined, *q*=1-*p*, *α* is the significance level (0.95) we wish to adopt, *r* is the number of replications used in the DCE (=12), and *a* is the precision with which we want the estimated p from a sample of size n around the true mean. We take *a* to be 0.1, meaning that we want the mean estimate of *p* from the sample to be at most ±10% from the true mean. With all of this defined, we have that

$n\geq\frac{0.8}{12(0.2)(0.1)^{2}}\left[\Phi^{-1}\left(\frac{1+0.95}{2}\right)\right]^{2}\approx128$respondents

In comparison, our actual sample size is N=604 respondents, which is about 4.7 times larger than indicated by Louviere, Hensher and Swait (2000). So, despite the simplifications that underlie the Louviere, Hensher and Swait (2000) method, it would seem to indicate that we have a reasonably comfortable cushion in terms of reliable estimation of the aggregate proportion being implicitly estimated by the choice model. Admittedly, the approximate method above is not based on minimizing a function of standard errors of estimated parameters, but it is strongly indicative that the sample size we ended up with is capable of providing reliable estimates based on a true outcome proportion of 0.20.

Also taking Orme’s rule of thumb (2010) into account,

$$N\geq\frac{500c}{at}$$

where N is the number of respondents, c is the maximum number of levels per attribute (in our case 19 levels), a is the number of alternatives per task (in our case three alternatives), and t is the number of choice tasks (in our case 12 choice tasks per respondent), our sample size of N=604 respondents is about 2.3 times larger than suggested. This further supports our conclusion above that we have a reasonably comfortable cushion in terms of reliable estimation of the aggregate proportion being implicitly estimated by the choice model.

*References*

de Bekker-Grob EW, Donkers B, Jonker MF, Stolk EA. Sample Size Requirements for Discrete-Choice Experiments in Healthcare: a Practical Guide. *Patient*. 2015;8(5):373-384. doi:10.1007/s40271-015-0118-z

Louviere J, Hensher DA and Swait JD. Stated Choice Models: Analysis and Application. *Cambridge University Press.* 2000. DOI: 10.1017/CBO9780511753831

Orme, B. Sample Size Issues for Conjoint Analysis, in Getting Started with Conjoint Analysis: Strategies for Product Design and Pricing Research. *Research Publishers, LLC.* 2010
