# Supplementary material for: Identifying the impact of social influences in health-related discrete choice experiments
Source: PLoS One. 2022 Oct 19;17(10):e0276141. doi: 10.1371/journal.pone.0276141 (PMC9581381; doi:10.1371/journal.pone.0276141)
Supplement: S3 Appendix — (DOCX) [file pone.0276141.s003.docx]

**S3_Appendix: Survey and Sample**

The online panel companies eliminated incomplete surveys before transmission of data, since their reward schemes did not pay respondents for incomplete surveys. A priori, it was expected that data quality would be high, particularly because of the high degree of involvement and motivation that mothers have around the topic of childhood vaccination. So it is not surprising that data checks for random choices indicated no evidence for this phenomenon. In fact, the trimmed mean completion time for the survey is 26 minutes (10% top/bottom trim), very much in line with our a priori expectations given length and complexity of the survey instrument. In addition, we note here that an oft used data quality test is to examine respondents’ tendency to “stick” with a given alternative in the choice task, e.g., choosing left-most alternative in all tasks. In our labelled DCE, such an exclusion criterion would inappropriately eliminate the majority of our data. We consider this test generally inappropriate, but particularly so in our case due to the fixed nature of the recommended schedule and opt-out alternatives, and the *a priori* high likelihood of the respondent population to follow the recommended schedule for their children.
