# Supplementary material for: Identifying the impact of social influences in health-related discrete choice experiments
Source: PLoS One. 2022 Oct 19;17(10):e0276141. doi: 10.1371/journal.pone.0276141 (PMC9581381; doi:10.1371/journal.pone.0276141)
Supplement: S4 Appendix — (DOCX) [file pone.0276141.s004.docx]

**S4_Appendix: Integrating multiple influencers into an ordered latent class choice model**

We express the unconditional probability that alter­native *j*∈*C*, *C* a choice set, is chosen through the probability expression

$P_{j}=\sum_{s=1}^{S} P_{j|s}W_{s}$ , [Eq. 1]

where *s* is the index for latent classes, *S* is the number of latent classes, *P_i|s_* is the conditional probability of choice given membership in class *s*, and *W_s_* is the likelihood of belonging to class *s*. We describe these submodels in sequence. (Note: the index “n” for decision maker should be understood to be present throughout. We omit it for the sake of clarity.)

*The Conditional Choice Model*

To account for social network influences and modelling our DCE data by assuming a utility maximisation decision process, we use the choice model formulated by Swait & Marley^42^ extended for multiple influencers (see equation 1) to estimate the choice probability for childhood vaccination schedule alternative *j*. Specifically,

$p_{j|s}=\frac{\exp\left(\lambda_{s}V_{j|s}+\sum_{g\in G} \alpha_{g}^{s}\ln q_{gj}\right)}{\sum_{i\in C} \exp\left(\lambda_{s}V_{i|s}+\sum_{g\in G} \alpha_{g}^{s}\ln q_{gi}\right)}, \forall j\in C$ [Eq. 2]

is the probability (*p_j|s_*) that a respondent belonging to class *s* chooses alternative *j* in choice set *C*, a function of the class-specific utility (*V_j|s_*) for alternative *j* compared to all alternatives (*i*) in choice set *C*; this utility is explained in terms of the childhood vaccination schedule attributes, scale factor *λ_s_*, and the class-specific strength ($\alpha_{g}^{s}$) of each alternative-specific influence (*q_gj_*) of influencer *g*. For identification, the scale factors are restricted to unity and the *α*’s estimated – in general, one parameter in the set {*λ*,*α_1_*,…,*α_G_*} must be fixed for the identification of the remaining parameters in the set.

The social influence component of the model is described by the term $\sum_{g\in G} \alpha_{g}lnq_{gj}$ in equation 2, which gathers together the aggregate impact of the perceived recommendation distributions for the key influencers identified by the decision maker. These distributions, which we measured as likelihoods of recommendation, are provided by the decision maker, and reflect their perceptions of what the influencers would like the decision maker to decide vis-à-vis the child’s vaccination schedule. The *α*’s capture the impact of the key influencer’s desires with respect to vaccination relative to the aggregate impact of the utility of the alternative (*V_j|s_*).

*The Class Membership Model*

Suppose there are latent underlying factors which define how preferences V_j|s_ are discriminated. Almost universally, models of discrete categorization of preferences rely on the assumption that there are multiple (say K) such factors, and in the resulting space K-space the segments or classes are categorical in that the class membership factors are orthogonal to one another. The likelihood of membership in a class *s* is essentially a volume in K-space.

In our model, we wish to discriminate preferences of mothers for their children’s vaccination as a function of a single dimension, defined by the WHO_SAGE vaccine hesitancy scale. Its stochastic representation here is given by

$Y=WHO\_SAGE + \nu$ [Eq. 3]

where -∞<ν<+∞ is a stochastic perturbation. Note that the parameter for WHO_SAGE is constrained to unity.

Define a latent class indicator $\Sigma$, which takes on values in the set {1,...,*S*}, and is related to *Y* as follows:

$\Sigma=\left\{ \begin{matrix} 1 \\ 2 \\ \vdots\\ \text{S} \end{matrix}\right. \begin{matrix} \text{Y}\leq\tau_{1} \\ \tau_{1}\leq\text{Y}\leq\tau_{2} \\ \vdots\\ \tau_{S-1}\leq\text{Y} \end{matrix}$ [Eq. 4]

where the *τ_s_*, *s* = 1,...,*S*-1, are cutoff parameters to be estimated that define the ranges of *Y* that lead to classification into each latent class. For S classes, only *S*-1 cutoff parameters are needed to define them, and one of them needs to be held constant to identify the remainder. (See Greene 2018 for a definition of and motivation for using ordered latent variable models.)

Since *Y* is a random variable, we must assume some distribution to describe the stochastic term *ν* so that the probabilities $W_{s}=\Pr(\Sigma=s)$, *s* = 1,...,*S*, may be calculated. Assuming that *ν* is independently logistic distributed across individuals and classes, its cumulative density function is

$G(\nu)=[1+\exp(-\nu)]^{-1}, -\infty<\nu<\infty.$ [Eq. 5]

Therefore,

$W_{s}=\Pr(\Sigma=s)=\left\{ \begin{matrix} G(\tau_{1}-\bar{Y}) \\ G(\tau_{2}-\bar{Y})-G(\tau_{1}-\bar{Y}) \\ \vdots\\ 1-G(\tau_{S-1}-\bar{Y}) \end{matrix}\right. \begin{matrix} s = 1 \\ s=2 \\ \vdots\\ s=S \end{matrix}$ [Eq. 6]

where $\bar{Y}=E_{\nu}(Y)=WHO\_SAGE$ (see expression 3).

Note that expression (6) imposes an ordinal relationship among the latent decision classes: membership in higher indexed classes implies higher values of *Y*, and vice-versa. The cutoffs define ranges on the single latent factor that correspond to different sequential (or ordered) classes, as interpreted from the configuration of relative attribute importances contained in the associated taste vectors *β_s_*, *s* = 1,...,*S,* where *V_j|s_=β_s_*X_j_*. In other words, *Y* is an index or score, and based on the notion that as the elements changing *Y* (essentially, vaccine hesitancy score WHO_SAGE) increase it, decision maker preferences change. More specifically, as vaccine hesitancy increases, preferences may change.

The above formulation allows for the error-in-variables possibility in the WHO_SAGE scale through the stochastic term *ν*. The classification function is underpinned by a latent variable that allows for its own logistic error term, which thus allows for errors-in-variables that are assumed orthogonal to the stochastic terms in the conditional utility function. That is, we construct a classification mechanism which endogenously allows for possible errors-in-variables (and other biases) in the WHO_SAGE scale through the formative estimator of the underlying vaccine hesitance scale.

References:

Greene, William (2018), *Econometric Analysis*. Eighth Edition, New York: Pearson.
