# Supplementary material for: Identifying the impact of social influences in health-related discrete choice experiments
Source: PLoS One. 2022 Oct 19;17(10):e0276141. doi: 10.1371/journal.pone.0276141 (PMC9581381; doi:10.1371/journal.pone.0276141)
Supplement: S5 Appendix — (DOCX) [file pone.0276141.s005.docx]

**S5_Appendix: Discrete Choice Experiment Analysis**

The systematic utility function *V_j_* for the designed (middle) alternative in our study was comprised of a linear-in-parameters coding of the delayed schedule attributes described in Section 2.1 of the manuscript. Alternative Specific Constants (ASC’s) were included for the Official Recommended and Delay/Split alternatives, relative to the No Vaccination alternative. Because of the moderate sample size, but particularly because of the higher relative importance of social influences vis-à-vis schedule attributes, it was found to be empirically necessary to simplify the coding of several attributes. For example, the number of injections for the Delayed alternative varied from 1 to 19 over a 12-month period. We found it necessary to simplify the coding to {more than, same as, less than} the injections required in the Official recommended alternative; the “same as” level is used as the base (i.e. reference level), thus reducing from 18 parameters to be estimated to two for model identification. The same occurred for number of visits over a 12-month period. When the choice model considered both childhood vaccination attributes and key influencers, a very high model fit was reached.

The magnitude of the class assignment threshold *τ* indicates the cut-off value of the WHO vaccine hesitancy scale separating two (consecutive) classes. That is, the probability to belong to a specific class is determined by the WHO vaccine hesitancy scale value. See S4_Appendix for more details. To be more precise, a clear threshold *τ*-value identified by the OLCM will act as the cut-off value between class #1 and class #2. Thus, a WHO vaccine hesitancy score that was higher than the cut-off value means the respondent belonged to class #1, while a WHO vaccine hesitancy score lower than the cut-off value means the respondent belonged to class #2. To simplify the use of the hesitancy scale, for our purposes we simply used unit weights for the scale items, duly signed to take into account whether the item is pro- or con-hesitancy. Simply put, the higher the score, the higher the perceived hesitancy.
