# Supplementary material for: Identifying the impact of social influences in health-related discrete choice experiments
Source: PLoS One. 2022 Oct 19;17(10):e0276141. doi: 10.1371/journal.pone.0276141 (PMC9581381; doi:10.1371/journal.pone.0276141)
Supplement: Attachment — Submitted filename: Response to Reviewers.docx [file pone.0276141.s007.docx]

**Response to Reviewers**

We thank the reviewers and the editor for the evaluations, positive words and the valuable comments made, which we used to improve our manuscript. Please find a point-by-point response to each comment presented below.

**Reviewer #1**

*“This manuscript reports a discrete choice experiment (DCE), designed to investigate the impacts of vaccine schedule characteristics and social network influences on decisions made by pregnant women regarding childhood vaccination. The results indicate that these decisions are almost entirely socially driven, providing insights that are both vital and timely as we continue to navigate our way through a global pandemic. This is an exceptional study, with important implications for public health policy, DCE methodology, and the recognition of social influences on individual choice behaviour within health economics. I have few comments, most of which are aimed at making the manuscript more accessible to readers who are unfamiliar with the more technical aspects of DCE methodology. This is with a view to attracting as large an audience as possible for this very important work.”*

*“Specific points:”*

1. *“p6, lines 124-136: It might be clearer to present these as bullet-points or in a table.”*

**Response:** We now present the attributes and alternative specific levels in a table (see Table 1)

2. *“p6, line 146: Please insert “(MNL)” after “multinomial logit” – this is because the abbreviation MNL appears on p11 (line 213) without having been defined previously.”*

**Response:** Done (see 2.2 Discrete choice experiment (DCE), p.8)

3. *“p10, lines 179-184: It would be useful to know a little more about the aims and basic designs of the Zingg et al, Larson et al and Holt et al studies, to explain why these were used as the sources for the questions about vaccines, vaccinations and susceptibility to social influences.”*

**Response:** We added more details now why Zingg et al, Larson et al and Holt et al studies were used as the sources for the questions about vaccines, vaccinations and susceptibility to social influences (see 2.3 Survey and sample, p.12). In more detail, using Mokken scale analyses and a test-retest analysis, Zingg et al developed a one-dimensional knowledge scale about vaccination with good psychometric properties (substantial correlations between knowledge and willingness to vaccinate). Their scale is a useful tool for detecting misconceptions in a time when new media such as the Internet are highly accessible to most people, and their scale is well suited for research examining group differences (Zingg et al). Larson et al developed a matrix of the determinants of vaccine hesitancy using a mixed methods design. Their matrix mapped the key factors influencing the decision to accept, delay or reject some or all vaccines under three categories: contextual, individual and group, and vaccine-specific. These categories framed the menu of survey questions to help diagnose and address vaccine hesitancy (Larson et al). Holt et al developed and validated an instrument to assess the perceived role of others in the health behaviour decisions of individuals. Their valid and reliable instrument will help answer some of the questions about the role of social networks and social influence processes on health behaviours and outcomes (Holt et al).

4. *“p11, lines 214-218: I think it would aid understanding for readers who are unfamiliar with latent class models (LCMs) if the underlying latent ordinal driver (the WHO vaccine hesitancy scale) were identified at this stage. In fact, the section that appears on p13 lines 257-262 could usefully appear here, as it provides a good explanation of why using a LCM is appropriate in this context.”*

**Response:** Thank you for this suggestion. We revised the manuscript accordingly (see 2.4. DCE analysis, p.14)

5. *“p11-p14, Section 2.4 DCE Analysis: I wonder whether this section may be a little challenging for readers who are unfamiliar with DCEs; although I appreciate that this is difficult to address, given that DCEs are inherently quite technical and specialist. In particular, the text that appears on p13, lines 250-256, is critical to understanding how data from the questions on key influencers is incorporated into the model. Is there a way that this could be simplified for non-specialists?”*

**Response:** We agree with the reviewer that this section might be challenging for readers who are unfamiliar with DCEs even though we had already strived to keep the econometric part as accessible as possible for non-econometricians and used words and examples instead of formulas where possible. To simplify the Methods section for non-specialists without running the risk of creating a black box for specialists, and taking all reviewers’ comments into account we did the following: (1) We added Table 1 to give a clear overview of attributes and alternative specific levels used; (2) We re-ordered the text to make it more reader-friendly (see section 2.2, pp. 7-8); (3) We removed the technical details and equations from the manuscript and put them in a supplementary material (see section 2.4, and S1_Appendix); and (4) We optimized the readability of Table 4.

6. *“p12, line 241: What does ASC stand for?”*

**Response:** Thank you for noticing that this abbreviation was not introduced yet. ASC stands for alternative specific constant. We added this term to the manuscript now (see 2.4. DCE analysis, p.16)

7. *“p22, lines 382-388: Would this fit better in the Discussion section?”*

**Response:** We agree that these sentences are better positioned in the Discussion section. We revised the manuscript accordingly (see 4. Discussion, p.30).

8. *“p25, lines 460-465: I can’t follow the point that is being made here.”*

**Response:** To make our point, we rephrased the lines as follow: ‘Our DCE outcomes and the outcomes of the direct survey question regarding how important the view of a certain key influencer was to the decision maker showed a reasonable level of convergent validity. That is, on the one hand, it showed that both methods led to the conclusion that the partner of the gravid woman had the largest impact on the maternal choice for childhood vaccination. On the other hand, it showed that who were the second and third important key influencers depend on the method used.’ (see 4. Discussion, p.31).

9. *“Finally, I thoroughly enjoyed reading this excellent manuscript. Thank you to the editors for assigning it to me. I’m looking forward to seeing this in print.”*

**Response:** It is really a pleasure to read that our work is appreciated and even enjoyed. Thank you so much for these supporting words and for your time.

**Reviewer #2**

*“A well-written paper reporting a well-conceived study with a very competently performed data analysis. I think it is a significant contribution. I suggest some minor changes:”*

1. *“The natural log is sometimes reported as "ln", others as "LN", it should be consistent”*

**Response:** The natural log is now consistently reported as “ln” throughout the manuscript, including Table 4.

2. *“Lines 353 and 372, surely it is not R-squared, but pseudo-R-squared”*

**Response:** Thank you for pointing at this. We revised the manuscript accordingly (see 3.2. DCE results, p.22 and Table 4).

3. *“In table 4, not sure what the scoring means and why it is reported”*

**Response:** To quickly review the classification model, membership in the classes is determined by responses to the 10 measurement items shown in Table 4, which are aggregated/summed together using the weights (±1’s) also shown in Table 4. Making these weights explicit serves two functions: 1) it makes explicit that some items have positive or negative valence; and 2) shows that these weights were not estimated from the data, but defined so as to allow direct and easy subsequent use of the classification model in any advisory tools that might be developed from our results. The items themselves were coded according to the coding scheme also included in the table under the heading “Responses on Likert scale (code for scoring function)”. The resulting WHO vaccine hesitancy score (=weights * item responses) could range from -20 to +20, though empirically ranged from -20 to +15 (see Table 3). The breakpoint between the preference classes is 1.1304 on the vaccine hesitancy scale. By making all the details of the score construction explicit, any user can apply the scoring mechanism above to determine class membership for a randomly selected mother in the target Australian sub-population, and thence have preference estimates for that mother without having to conduct a DCE. We have added this description in section 3.2 DCE results, pp. 22-23.

4. *“Line 444 "to get uptake of the childhood vaccination", can this be written better?”*

**Response:** This part of the sentence has been rewritten as “to increase the uptake of childhood vaccination (see 4. Discussion, p.30).

5. *“Lines 449-451, not clear what is intended here”*

**Response:** We agree that the original sentence was unnecessarily vague. We rephrased the sentence as follows: “That said, our study still showed that the DCE method is relevant here – even if the attributes of the vaccination were not the strongest drivers of stated choices for childhood vaccination in Australia - but also showed that DCE could benefit from extension.” (see 4. Discussion, p.30)

6. *“Importantly, some explanation should be provided about the model defined as "two-class ordered latent class model", I understand the panel, and I can see the MNL component, but it is unclear to me what the ordering is about, and how to interpret tau. What is the nexus between the (panel) probability of selections of the three vaccination options and the class membership, which appears to be linked to the ordering?”*

**Response:** Our desire to maximize econometric accessibility for a broad audience may have worked against communicating the details of the full modelling framework. We have therefore decided to detail the model fully in the S1_Appendix, which has been rewritten almost completely. We hope the expanded mathematical detail enhances your understanding (as well as that of other readers) of the ordered nature of the latent classes and the interpretation of threshold parameters.

**Reviewer #3**

*“This is a very interesting paper which explores the effect of social influence on preferences for childhood vaccination among pregnant mothers in Australia. The paper concludes that social influences are the primary driver of decision making beyond any features of the vaccine schedule and delivery. Exploring other factors which drive decision making beyond service features is critical for expanding the predictive ability of choice experiments for policy makers, particularly for policies such as vaccination where social norms play a large role in decision making. Identifying approaches to incorporate social influence econometrically is important. That being said, the paper has a few fundamental flaws: Firstly, the DCE methodology seems potentially flawed, primarily because of the large number of attribute levels and unclear sample size estimation, given the authors extensive experience in DCE methods and design, the authors should clarify in the limitations some of the issues related to the design and sample size and how this may have influenced the results of the DCE findings. It may well be that decision making is entirely socially driven, however if there were not sufficient participants or if there were cognitive difficulties in answering the DCE it would not be prudent to draw such strong conclusions. Therefore, the survey/DCE tool should be included in the supplementary materials, details of poor responses and internal validity checks should be detailed. The sample size is important, particularly because so many results are “not significant”. Second, the presentation of the methods and results sections in particular is very unclear. The methods, results and discussion are intermixed and therefore the reader must look for methods in the results section at times or find discussion points in the methods. The main results (Table 4) are not clearly presented and therefore make the results hard to interpret. The actual DCE findings are not discussed in any detail, presumably because little was “statistically significant”. Some methods sections are too long and should be presented more succinctly in the main paper with remaining econometrics included in supplementary materials. The authors and co-authors should review and make the methods and results sections as clear and succinct and well labelled as possible for a general readership to easily read and interpret. Given that this is not a health economics journal the authors should aim to make the paper readable for a general audience. Detailed comments follow”*

**Response:** We thank the researcher for the support and extensive feedback, which definitely helped us to transform our manuscript into a clearer and more reader friendly manuscript. See below our response to each detailed comment given.

Methods:

1. *“The authors state this is a mixed methods study, there does not appear to be a qualitative component to the study, literature review is a common component of DCE surveys. Suggest the authors drop the text regarding mixed methods (which have their own methodology) and restrict to a DCE survey, which is what appears to have been done.”*

**Response:** We now dropped the text regarding mixed methods and restrict to a DCE survey (see 2. Methods, p.5).

2. *“Rename the section 2.1 to “literature review and attribute selection””*

**Response:** Done.

3. *“Insert a table with final attributes and levels and reduce the text (lines 124-136 on page 6). Although a choice scenario example is presented in Table 1, it is not as easy to understand the design from visualizing only one choice task – both can be included in the paper or the current table 1 can go in supplementary materials.”*

**Response:** We followed the advice to add a table with final attributes and alternative specific levels and removed the corresponding text from the manuscript (see Table 1). The scenario example has been removed as this is now covered by making the DCE survey (see comment #10 of Reviewer #3) available for the reader upon request.

4. *“Section 2.2 – DCE appears to have 8 attributes in the text but 7 in the scenario. Also, section 2.1 suggests an attribute with 19 levels but section 2.2 shows 18 levels. Authors should review the manuscript for consistency of methods and results.”*

**Response:** Reading comment #6 of Reviewer #3, it seems the confusion regarding the number of attributes and levels is coming from the fact that we used a labelled DCE design without mentioning the term ‘labelled DCE survey’ in the manuscript explicitly. To improve the readability of our study and to avoid any misinterpretations from the number of attributes and levels, we now presented the attributes and alternative specific levels in one overview (see Table 1) and added the term ‘labelled DCE survey’ in the manuscript (see 2. Methods, p.5, and 2.1. Literature review and attribute selection, p.6). Moreover, we removed unnecessary technical details to adapt the manuscript better to the general reader and re-order the text (see section 2.2. Discrete Choice Experiment (DCE), pp. 7-8).

5. *“The number of levels for several attributes seems excessive and brings into question the overall design of the experiment. Attributes with 12, 16, 19 levels are unusual for DCE’s because of a) the inability to obtain balance in the design and b) the inability of the participants to choose between the levels that are very similar for example 17 versus 18 visits to for vaccination. Even with the use of Ngene and development of an efficient design and blocking, this methodology is questionable given that each respondent answered 12 choice tasks and sample size was relatively small. It appears that later these attribute levels were collapsed for this very reason. Please include some details in the limitations section regarding this potential design flaw and its implications for the results.”*

**Response:** As we used a labelled DCE design and a large design of 600 choice tasks, a fair level balance was reached (i.e., the correlation between attribute level combination in the design was kept low (<0.4)). That said, be aware that a (Bayesian) D-efficient design does not strive to reach perfect level balance but strives for an optimal choice design to be able the estimate robust outcomes. Also, the comparison between 17 versus 18 visits for vaccination took never place due to the labelled DCE design we used. Although each respondent answered 12 choice tasks, the design itself contained 600 choice tasks and had more than sufficient degrees of freedom to take preference heterogeneity into account. Our sample size of N=604 was higher than an average DCE in healthcare (see Watson, Becker, de Bekker-Grob. 2017 (reference #51) and was also higher than Orme’s rule of thumb suggested (N=264) (see reference #52). Namely,

$$N\geq\frac{500c}{at}$$

where N is the number of respondents, c is the maximum number of levels per attribute (in our case 19 levels), a is the number of alternatives per task (in our case 3 alternatives), and t is the number of choice tasks (in our case 12 choice tasks per respondent). In addition, if one develops a model with dummy or effects coding, one may empirically determine that subsets of them do not differ statistically. From this, one may aggregate the effects that are not significantly different, in an attempt to reduce model complexity and come up with a good, reduced form model rather than keep every possible parameter alive. That said, if dummy or effects coding is used and choice of a level is never made, it is not possible to identify the corresponding effect. Hence aggregation of the levels becomes mandatory to achieve identification. Nevertheless, like all quantitative studies, this study could have benefitted from a larger sample size to give even more detailed outcomes. We added these details in the limitations section now (see 4. Discussion, p.32).

6. *“It appears that a labelled design was used, it is worth naming the DCE as such in the methods.”*

**Response:** Thank you for this remark. Indeed, a labelled design was used. We agree with the reviewer that it is worth naming the DCE as such in the methods to be very clear from the beginning onwards. We revised the paper accordingly (see 2. Methods, p.5).

7. *“Could the authors please expand on the sample size estimation in the methods; although as noted, this is complicated for DCE’s – given the large design and the use of priors to develop the design, these same priors could have been used to estimate a sample size. Suggest the authors give more detail on the ideal sample size based on the design and priors/final estimates, even though affordability was a driver of the final sample size estimation. This will help to identify to what extend the analysis was limited by the samples size.”*

**Response:** Unfortunately, we cannot use formal parametric sample size calculation approach for DCE studies from de Bekker-Grob et al (2015, The Patient) (i.e. taking the DCE design and priors/final estimates into account) as that approach is for unlabelled DCE studies and MNL models only. However, we can use the general sample-size method given in Louviere, Hensher and Swait (2000) to provide a sample size estimate that does not take the priors into account, and so is far more conservative than would be the case otherwise. But nonetheless, it provides us with a value that is useful as a comparison point to the sample size that was actually collected (N=604). Consider that we are trying to estimate a (critical) multinomial probability of around *p*≈0.20, which is approximately the proportion of choices actually observed for the designed alternative in the DCE. The appropriate formula from Louviere et al. (2000) is expression (9.5):

$$n\geq\frac{q}{rpa^{2}}\left[\Phi^{-1}\left(\frac{1+\alpha}{2}\right)\right]^{2}$$

where *n* is the required minimum sample size, *p* has been defined, *q*=1-*p*, *α* is the significance level (0.95) we wish to adopt, *r* is the number of replications used in the DCE (=12), and *a* is the precision with which we want the estimated p from a sample of size n around the true mean. We take *a* to be 0.1, meaning that we want the mean estimate of *p* from the sample to be at most ±10% from the true mean. With all of this defined, we have that

$n\geq\frac{0.8}{12(0.2)(0.1)^{2}}\left[\Phi^{-1}\left(\frac{1+0.95}{2}\right)\right]^{2}\approx128$respondents

In comparison, our actual sample size is N=604 respondents, which is about 4.7 times larger than indicated by Louviere et al. So, despite the simplifications that underlie the Louviere et al. method, it would seem to indicate that we have a reasonably comfortable cushion in terms of reliable estimation of the aggregate proportion being implicitly estimated by the choice model. Admittedly, the approximate method above is not based on minimizing a function of standard errors of estimated parameters, but it is strongly indicative that the sample size we ended up with is capable of providing reliable estimates based on a true outcome proportion of 0.20.

Also taking Orme’s rule of thumb into account (see our response to comment #5 of Reviewer #3), our sample size of N=604 respondents is about 2.3 times larger than suggested. This further supports our conclusion above that we have a reasonably comfortable cushion in terms of reliable estimation of the aggregate proportion being implicitly estimated by the choice model.

8. *“Was any piloting or cognitive interviews conducted, please report on these tool development stages.”*

**Response:** A formal pilot study was not conducted. Informal piloting only was conducted with a small number of people; formal cognitive interviewing or think aloud approaches were not conducted.

9. *“Was the survey incentivized, assume so – this should be mentioned in the methods for the survey panel data.”*

**Response:** We did not incentivise the survey, but the panel companies had panellist reward systems that we had no control over. So, respondents were incentivized according to the standard reward scheme used by the two panel companies. We added these details to the manuscript (see 2.3. Survey and Sample, p.13).

10. *“Please include the original survey tool in supplementary materials.”*

**Response:** As we do not have screenshots of the DCE survey implementation, unfortunately we can only share the last draft of the DCE survey before it was sent to the (outsourced) programmer (see Appendix R.1 below). As we only can share the last draft of the DCE survey before it was sent to the programmer, instead of including the draft of the survey tool into a supplementary material, we decided - to avoid any misinterpretations - to add the following text to the manuscript: “The DCE survey is available upon request” (see 2.3 Survey and Sample, p.13). If the request is made by the interested reader, we can then also offer our help to explain the survey in more detail if needed. Obviously, we are more than willing to put the last draft of our DCE survey into a supplementary material if the editor is more favourable about that option.

11. *“How was internal validity determined – were any measures built into the design or explored afterwards, if so please describe - online surveys are notorious for poor responses.”*

**Response:** We did not build in any specific design features to “trap” poor responses. We relied very much on the high interest levels about the vaccination topic and the strong motivations of the population of Australian mothers to make their preferences known. These two factors explain data quality, in that topic involvement and importance lead to high quality data, while low involvement and/or low importance lead to low quality data in any environment, online or otherwise. As noted in our response to comment #17 of Reviewer #3, our data quality is high, despite the fact that the DCE indicates weakly determined preferences through the experiment. Notably, however, despite the lack of significance of most attributes, the vaccination behaviour of mothers is strongly explained by the social influences, which is our view, denotes a strong success. Following good practice, we have not created nor detected any serious problem with our well-considered labelled DCE. However, we have found that the outcome choice is explained by a different mechanism than attributes, namely, social influences.

12. *“The key influencer moderating characteristics scales and WHO vaccine hesitancy scale play a large role in the analysis and conclusions. Could the authors include the original scales and how they were analysed (scores generated from the original tool etc) prior to inclusion in the DCE modelling - in the supplementary materials.”*

**Response:** The WHO vaccine hesitancy scale we adopted was developed by Larson et al. (reference #17 in our bibliography). Details of the scale can be found in that reference. Note that our manuscript makes explicit in Table 4 the 10 measurement items from Larson et al. that are used in our hesitancy score (which we have termed WHO_SAGE). In addition, we show in that same table the ±1 scores that are applied to each item to calculate WHO_SAGE. So, to be crystal clear, we adopted the Larson et al. scale items, and employed signed unit weights as shown in Table 4 to calculate the vaccine hesitancy score used in the choice model. Unit weights were employed to make it easy for the model results to be employed by non-econometricians to categorize Australian mothers into the two preference classes we found.

13. *“Suggest section 2.4 be shortened and simplified for a general Plos One audience and that the detailed econometrics be included in the supplementary material to shorten this section and clarify the methods succinctly in the main paper.”*

**Response:** We have shortened section 2.4 substantially and put the detailed econometrics in a supplementary material. Additionally, we re-ordered the text in such a way that it should fit the general PLoS One audience better (see section 2.4. DCE analysis, and S1_Appendix).

14. *“It looks like the results are referenced in the methods eg. Table 4 in line 297 on page 11.”*

**Response:** We moved the corresponding sentences from the Methods to the Results section (see 3.2. DCE Results, p.26) and removed any further results referenced in the Methods section (see 2.4. DCE analysis, p.18).

15. *“Please name the “customer developed software” used for analysis.”*

**Response:** The expression in the paper is “custom developed software”, meaning that one of the authors developed problem-specific code from scratch. No “package” such as Stata, R, Julia, etc., was used. The actual code is in Fortran 77, and is proprietary to an organization in which one of the authors was present during development. A small adjustment to the text has been made in the paper: “… led to the decision to employ (after exploring a traditional MNL first) a panel ordered latent class model (OLCM) using custom-developed software in Fortran 77 (see 2.4. DCE analysis, p.14).

16. *“It appears that the methods for generating a WHO vaccine hesitancy scale cut-off are presented in the results. Please ensure that the methods for generating scale cut-offs are detailed in the methods sections (without presenting results in the methods section). It appears from the results that the WHO scale cut-off was developed based on the latent class membership – this should be detailed in the methods.”*

**Response:** The reviewer is right that the WHO scale cut-off was developed based on the latent class membership. We added these details to the Methods section now. In addition, we now explained the methods for generating the scale cut-off in the Methods section – instead of the Results section – without presenting results in the Methods section (see 2.4. DCE analysis, p.18). We have also added more details about the classification model into the S1_Appendix.

Results

17. *“3.1 Please include details on poor; incomplete responses and internal validity checks/random responses”*

**Response:** Incomplete/abandoned surveys were excluded from analysis, done directly at source by the online panel company. Among retained respondents, all items related to the focal measurements (choices, perceptions of influences, and WHO_SAGE) were mandatory for respondents, so these presented no missing items for analysis. As we previously noted in the manuscript (section 3.2), 78% of mothers always chose the recommended schedule, 19.4% traded between alternatives, and less than 3% always chose delayed schedule or opt-out. That is to say, around 80% of the sample displayed fixed choice pattern which we deemed eminently reasonable. So, checking for “straight-lining” is not a sensical test of data quality in our context. We found no evidence of “random” decision making. This is not surprising in that the topic of childhood vaccination is highly involving and motivating for mothers, as the statistics in the prior bullet point attest. No completion-time-based restrictions were imposed, which resulted in a trimmed mean of 26 minutes completion time, which was in line with our expectations given the length and complexity of the survey. We reflect these considerations as follow in our revised manuscript: “We utilized all respondents that completed (i.e., did not abandon) the survey before it was closed off at the end of the data collection period. The online panel companies eliminated incomplete surveys before transmission of data, since their reward schemes did not pay respondents for incomplete surveys. A priori, it was expected that data quality would be high, particularly because of the high degree of involvement and motivation that mothers have around the topic of childhood vaccination. So it is not surprising that data checks for random choices indicated no evidence for this phenomenon. In fact, the trimmed mean completion time for the survey is 26 minutes (10% top/bottom trim), very much in line with our a priori expectations given length and complexity of the survey instrument. In addition, we note here that an oft used data quality test is to examine respondents’ tendency to “stick” with a given alternative in the choice task, e.g., choosing left-most alternative in all tasks. In our DCE, such an exclusion criterion would eliminate almost 80% of our data. We consider this test generally inappropriate, but particularly so in our labelled DCE case due to the fixed nature of the recommended schedule and opt-out alternatives.” (see 2.3. Survey and Sample, pp. 13-14)

18. *“Please add footnote to table 2 regarding how to interpret WHO vaccine hesitancy”*

**Response:** We now added the following footnote: ‘Unit weights for the WHO vaccine hesitancy scale items were used, which can lead to a maximum total vaccine hesitancy value range from -20 to +20; the higher the score, the higher the perceived vaccine hesitancy’ (see footnote Table 2).

19. *“On page 20 line 362 – 366 the authors present results related to adherence and uptake of vaccination schedules – it is unclear how these data can speak to adherence or uptake rates. Please remove this section from the results or rephrase.”*

**Response:** To avoid any misinterpretation, we now removed this section (see 3.2. DCE results, p.23).

Table 4

20. *“It is unclear what all the results in the table represent – some sections have percentages other have utilities”*

**Response:** To improve the readability, the terms ‘class probability’ and ‘coefficient’ were added to the table. Taking comment #19 of Reviewer #3 into account, we removed the cells concerning ‘Segment Conditional Choice Distribution’ (see Table 4).

21. *“It is unclear what is being presented in the “Classification Scoring”*

**Response:** Please see our response to comment #3 of Reviewer #2, which we believe addresses your question. See also the addition of footnote #1 in the revised manuscript.

22. *“Function: WHO_SAGE Vaccine Hesitancy Scale “Please can the authors clarify this section of the table it is unclear what the reader is looking at.”*

**Response:** Please see our response to comment #3 of Reviewer #2, and footnote #1.

23. *“Consider breaking this table up into multiple tables with clear column and row headings and appropriate footnotes. Or organize the table so that the reader can quickly distill what results are being evaluated and how to interpret them.”*

**Response:** After careful consideration, we decided to organize the table so that the reader can quickly distil what results are being evaluated and how to interpret them. See also our responses to comments #20, #21, #22 and #24.

24. *“Authors should consider naming latent class 1 and latent class 2; this will help the reader to quickly understand which group is being referred to in the results throughout eg. “vaccine hesitant” vs “vaccine accepting” or something along these lines.”*

**Response:** We thank the reviewer for this suggestion. We revised Table 4 accordingly (see Table 4).

25. *“Consider placing the “Perceived allocation of Key Influencer to the vaccination option: [0,100]” section in a separate table and clearly label what exactly is being reviewed in this section.”*

**Response:** For each Key Influencer (KI) identified by a respondent, the respondent was asked ‘For <key influencer type X>, please tell us how likely you believe they would be to recommend each vaccination option by allocating 100 points across the three options for this person, with more points meaning that they are more likely to recommend that option’. Responses to these questions are the Perceived Allocation of Key Influencer variables used in the model and identified in Table 4. Thus, these quantities are the ln(*q_gj_*) used in the conditional choice model (expression 2) in the S1_Appendix. Our labels for these data are taken from the survey, and were previously described in Section 2.3. We have added the *q_gj_* symbol to Table 4 to better connect the contents of the table to the updated model description in the supplement.

26. *“Tables should be interpretable on their own, without having to comb through the methods and results to aid interpretation.”*

**Response:** We completely agree with the reviewer. We hope we succeeded in this after revising all tables, except for Table 3.

27. *“Authors should present either linear or quadratic results in the table based on the best fit to the data, this should be specified consistently for all attributes modelled continuously.”*

**Response:** We believe the model should be presented in its fullest identifiable form, which is that presented in Table 4, rather than the reduced form model. We respectfully demur to follow your guidance.

28. *“Please include confidence intervals for utilities. If space constraints an issue, reduce decimal places”*

**Response:** We have provided p-value ranges for the coefficients on purpose. We respectfully disagree to include confidence intervals for utilities. This would make the table quite difficult to read even if we reduce decimal places, simply because there are so many coefficients, each with a confidence interval (!). To be honest, we tried this already for the original version, it was and still would get super messy. An alternative is to develop a version in a spreadsheet that can be obtained from the authors, upon request. Depending on the editor, we can deliver a messy Table 4 with confidence intervals or a spreadsheet with confidence intervals as a supplementary document.

29. *“The authors highlight that it is surprising that the attribute features had little influence on overall decision making and that the main decision drivers were related to social influences. Given that it is uncertain whether the sample size was sufficient to establish main utilities or latent classes, suggest the authors first explore the potential limitations of the sample size before making such claims. In addition, the authors identify early in the results that 19.4% of participants were willing to trade between alternatives, suggesting that some features of the scenarios must have influenced their decision making; could the authors reflect on this in the paper.”*

**Response:** See our response to comments #5 and #7 of Reviewer #3. The feature of our choice tasks that result into that 19.4% of the participants were willing to trade between alternatives had to do with the labelled DCE design. We deliberately chose to mirror the real choices that parents would be making with respect to vaccination – a fixed schedule, as per recommended (A); a variable schedule with possible delays/splits (B), and a fixed no vaccination schedule (C) – with only schedule B varying in each question. In each choice task, respondents were asked to opt for the alternative that appealed most to them. The inclusion of labels might have played a significant role in individual choices. That is, although the labelled DCE was highly likely more suitable to explain real-life choices than an unlabelled DCE, the consequence might be that the labels have reduced the attention respondents give to the attributes (de Bekker-Grob, Hol, Donkers et al. 2010. Value in Health; see reference #18) and hence trading off the alternatives in our labelled DCE survey.

30. *“This section below starting on line 384 on page 22 in the results, should be in the discussion, not the results section: “Given the authors’ prior experience with DCEs in health, marketing, transport and environmental economics, this is an unusual result since theory would suggest that attributes are the strong drivers of choices. Instead, this particular DCE is demonstrating that social influences might be a strong explanator of behaviours in certain contexts. This observation should be strongly motivating for further research in social influences on individual choices, calling for further empirical work and choice paradigm evolution.”*

**Response:** We agree that these sentences are better positioned in the Discussion section. We revised the manuscript accordingly (see 4. Discussion, p.30).

31. *“Can the authors postulate potential reasons for some inconsistencies in the influence of partners. Table 4 shows partners playing a large role in main preferences but not for the latent class groups or not in combination with vaccine knowledge. It appears that the effect of partners varies across the analyses. Given that this is one of the strongest conclusions of the paper it would be helpful for the authors to fully interpret all the data presented related to the influence of partners across the various analyses, not only those that were statistically significant.”*

**Response:** One pattern that jumps out is that Class #1 (vaccine hesitants) has most KI’s being significant. That may be indicative that members of this class are more likely to seek out and adhere to supporting information in their social circle, and conversely, reject disconfirming information. In contrast, Class #2 (vaccine prone) has comparatively few significant KI’s. Perhaps they are more confident/sure in their beliefs, hence depend less on preferences? We are hesitated to “stretch” the interpretation of the data beyond what the model is capable of discerning.

32. *“Although the results of the DCE were not statistically significant, the authors should present some narrative about the findings for the individual attributes and their levels, even if only non-significant trends are seen in the data. Currently there is no discussion of the actual DCE results.”*

**Response:** Like our response to comment #31 of Reviewer #3, it is perilous to draw conclusions or speculate much on statistically weak results. We therefore respectfully disagree to present some narrative about the findings for the individual attributes and their levels, even if only non-significant trends are seen in the data.

33. *“It is also hard to interpret the results when the final DCE design is not presented, it would be good to see what levels were presented for each attribute. For example, it appears that the “chance of your child getting a vaccine preventable disease” was important to participants though potentially not statistically significant. Confidence intervals may also highlight if the non-significant utilities are more around sample size than as the authors conclude that only social influence drives decision making”*

**Response:** See our responses to comments #3 and #28 of Reviewer #3.

Discussion/conclusion

34. *“Please expand the limitations to include potential issues with the DCE design/sample size/ online preference elicitation method. Please temper the strengths of the conclusions considering the possibility of flaws in the DCE design eliciting preferences for vaccine features. For example, it is hard to imagine that the risk of a child developing vaccine preventable illnesses is not important at all in decision making, which seems to be what the discussion is saying. It is more likely that it important but that social influences might be a stronger driver, for example.”*

**Response:** See our responses to comments #5, #7 and #11 of Reviewer #3. Equivalently, the social influences are an indirect way for the respondent to encapsulate and summarize the vaccine-related information garnered by de respondents’ KI’s. So the specific attributes are far less important in explaining attribute preference, which is instead summarized by perceptions of KIs recommendations. Our model gives the attributes the same importance in describing choice outcomes as it does the KI’s. However, the KI’s come out being the sole explanator. That is what makes these results so important. Hence, making a decision through the exercise of one’s own preferences is not the one and only way. However, our results are suggesting that there is another (social) mechanism for decision making in this domain, which is to listen to and be guided by the trusted people in your life. These sources also want the best for one’s child, so their opinion counts heavily, perhaps beyond one’s own preferences.

**Editor**

1. *“In particular, one of the reviewers had serious concerns about how the experimental design was conducted and how the results were reported (reviewer 3).  As concerns the experimental design, you should provide more information about the prior-setting procedure (i.e expert judgement process) and to what extent the assumed priors reflect the actual estimates.”*

**Response:** It seems that the misunderstanding about the experimental design was completely driven by the fact that we were not clear from the beginning that we conducted a labelled DCE design. We revised the manuscript substantially to not only reduce this misunderstanding but also to increase the readability of the paper and make it more suitable to the general PLoS One reader. See our responses to comments #4 - #16 of Reviewer #3.

2. *“Moreover, you should clarify the important inconsistencies that exist between the stated number of attributes/levels (2^3 x 4^1 x 5^1 x 10^1 x 16^1 x 18^1), what you  declared to consider as attributes/levels (i.e  "the number of injections over 12 months (levels: 0, 1,...,19)" have 20 levels;  visits to the clinic for vaccinations over 12 months (levels: 0, 1,...,16) have 17 levels; the chance the child will get a vaccine preventable disease (levels: 1/1000, 2/1000,…,12/1000) have 12 levels); and the way you included in the model: some attributes are considered as linear, (with quadratic term); an attribute had the ln transformation. I see several sources of unexplained arbitrariness in the model estimate. For instance, you declared that  the* 18  parameters  of "number of injections  attribute" have been reduced to two for model identification*" and the same has been done for # Clinic Visits Compared to Recommended Schedule. Authors should clarify If the DCE has been performed (and the related priors) by considering the parameter number of injections as "linear / continuous attribute " such as occurrences of side effects per 100 children, as 19 level attribute or 3 levels attribute.”*

**Response:** The actual design was obtained using the number of attributes/levels indicating in the factorial calculation in your comment. Once the data were collected, it was found that certain levels of attributes were never chosen, which leads to an inability to identify a dummy or effects coded specification arising straight from the design. The identification problem was mitigated by recoding certain of the design variables in a continuous fashion. This can be understood as a reduced form model based on a design that could identify a more complex model if the choices were differently distributed over the levels of attributes like “number of injections” and “number of clinic visits”. In our case, this simplification is driven by lack of variation in the choice variable when viewed from the perspective of certain attributes levels. This is no different than having a 4-level price variable, and having no one choose the alternative when the price is highest. The same kind of simplification would have to be made to the model. We give more attention to this issue in the revised manuscript. See our response to comment #5 of Reviewer #3.

3. *“The choice of an "ordered model" with two cut-off (3 levels) is unclear. Authors stated that "In each choice task, respondents were asked to opt for the alternative that appealed most to them. " Ordered model can be used in rated based tasks, or in ranking, while authors indicated in the text a choice-task.”*

**Response:** Our prior manuscript made this explanation difficult to understand, so we have tried to improve this explanation. A detailed specification of the model is now included in the S1_Appendix. Specifically, what is ordered in the model is not the alternatives in the choice set C (i.e., alternatives, ratings, or rankings), but instead, the preference classes are ordered. Assume there is a latent variable -∞<Y<∞ that undergirds/defines preference heterogeneity, and that the preference adoption process is such that lower Y’s define preferences different from higher Y’s. The class membership process, therefore, happens on the real number line Y and identification of preference classes requires identifying ranges on the Y latent variable. That is, preference class 1 is associated/defined by Y≤τ_1_, class 2 by τ_1_<Y≤τ_2_, …, and class S by τ_S-1_≤Y<∞. Thus, the classes are ordered, but the choices being predicted are still categorical. Again, please refer to the S1_Appendix for more details.

Journal Requirements:

4. *“When submitting your revision, we need you to address these additional requirements. Please ensure that your manuscript meets PLOS ONE's style requirements, including those for file naming. The PLOS ONE style templates can be found at* [*https://journals.plos.org/ plosone/s/file?id=wjVg/PLOSOne_formatting_sample_main_body.pdf*](https://journals.plos.org/%20plosone/s/file?id=wjVg/PLOSOne_formatting_sample_main_body.pdf)  *and https://journals. plos.org/plosone/s/file?id=ba62/PLOSOne_formatting_sample_title_authors_affiliations.pdf*

**Response:** The marked-up and unmarked versions of the manuscript for resubmission have been named “Revised Manuscript with Track Changes” and “Manuscript” as required. The supplementary material file has been named “S1_Appendix”. The authors believe the rest of the manuscript meets PLOS ONE’s style requirements, including font, headings, spacing, and line numbering.

5. *“Thank you for stating the following in the Acknowledgments Section of your manuscript: “Grant support was from Australian Research Council Discovery Project DP140103966 to the third author, and from The Netherlands Organisation for Scientific Research (NWO-Talent-Scheme-Vidi-Grant No. 09150171910002) to the first author. The funders had no role in the study design, the collection, analysis and interpretation of data, in the writing of the manuscript or in the decision to submit the manuscript for publication.” We note that you have provided additional information within the Acknowledgements Section that is not currently declared in your Funding Statement. Please note that funding information should not appear in the Acknowledgments section or other areas of your manuscript. We will only publish funding information present in the Funding Statement section of the online submission form. Please remove any funding-related text from the manuscript and let us know how you would like to update your Funding Statement. Currently, your Funding Statement reads as follows: “Grant support was from Australian Research Council (*[*https://www.arc.gov.au/*](https://www.arc.gov.au/)*) Discovery Project DP140103966 to JS, and from The Netherlands Organisation for Scientific Research (*[*https://www.nwo.nl/en*](https://www.nwo.nl/en)*) (NWO-Talent-Scheme-Vidi-Grant No. 09150171910002) to EBG. The funders had no role in the study design, the collection, analysis, decision to publish, or preparation of the manuscript.”Please include your amended statements within your cover letter; we will change the online submission form on your behalf.”*

**Response:** We now removed the funding information from the Acknowledgements Section. We confirm that the original Funding Statement we had, is still correct. No adaptations are needed.

6. *“We note that you have stated that you will provide repository information for your data at acceptance. Should your manuscript be accepted for publication, we will hold it until you provide the relevant accession numbers or DOIs necessary to access your data. If you wish to make changes to your Data Availability statement, please describe these changes in your cover letter and we will update your Data Availability statement to reflect the information you provide.”*

**Response:** Thank you for noticing this issue. We are happy to confirm that we are allowed to make all data necessary to replicate our study’s findings publicly available without restriction. We revised the cover letter and online system accordingly: "The data underlying the results presented in the manuscript are available from the Erasmus Choice Modelling Centre website via the following URL: https://www.erim.eur.nl/erasmus-choice-modelling-centre/resources/datasets/ "

**Appendix R.1: The DCE survey before it was sent to the (outsourced) programmer**

Parental Choices about Childhood Vaccination

You are invited to take part in this study. Please read this Explanatory Statement in full before deciding whether or not to participate in this research. If you would like further information regarding any aspect of this project, you are encouraged to contact the researchers via the phone number or email address listed above.

**What does the research involve?** 
The aim of this study is to gain understanding about the factors influencing mothers when they’re deciding about vaccinations for their children.

It involves answering an anonymous online survey, which will take around 20 to 30 minutes. In the survey you will be asked to make choices between alternative childhood vaccination schedules, and also to answer some questions about yourself.

**Who is asked to take part?** 
Women who are currently pregnant.

**Consenting to participate in the project and withdrawing from the research** 
By continuing to the online survey by pressing “>>” below, you are providing your consent to participate in this study. At any stage whilst completing the survey, you are free to change your mind and stop the survey. However, once you have submitted your responses at the end of the survey, it will not be possible to withdraw your data because the data is anonymous. If you do not wish to participate, you do not need to take any further action.

**Possible benefits and risks to participants** 
We do not anticipate any risks to you from participating in this survey.

**Storage of data** 
We will securely store and manage the data, and only the research team will have access to the data. The data will be stored in an anonymous way, and therefore your answers are not linked back to any identity.

**Results** 
The results of our analyses will be available in their published form after the study is completed. Because of our anonymity promise and the anonymity of the data collected, under no circumstances will results enable identification of any participant.

**Complaints** 
Should you have any concerns or complaints about the conduct of the project, please contact…[name + affiliation]

Thank you for participating!

Please click " >> " to continue.

**Section 1 – Qualification for participation**

Before continuing on to the body of the survey, we’d like to ask a few preliminary questions to make sure you’re qualified to participate.

1.1. My age is ____ years.

1.2. I am pregnant at this time. Yes/No

Programmer: If Q1.3=yes, go to BBB. // qualifies

Else Terminate with “We’re sorry, but you do not qualify to participate in this study. Thank you for your time.”

BBB: // Qualifies, continue

1.3 Approximately how far along is the pregnancy? ________weeks along

“>>” to Continue

Section 2 – Alternative Vaccination Schedules

On the following pages, you will be shown 12 scenarios that describe a) the current vaccination schedule recommended by the National Immunisation Program for children up to 12 months of age, and b) other hypothetical schedules that vary in terms of whether some vaccines are delayed, or whether combination vaccines are split into their different components or different visits to your GP or child health nurse. The consequences of these vaccination delays or vaccine splitting are described in terms of

- The number of injections your child would receive over the 12 months
- The number of clinic visits for vaccinations needed over the 12 months
- The chances your child might get a vaccine-preventable disease
- The chance of minor side effects from each set of vaccines (for example, mild temperature, irritability, pain at the injection site)
- Implications for formal childcare arrangements as a result of vaccination status
- Implications for family assistance payments as a result of vaccine status

The following page shows you an example scenario – each scenario presents the current vaccination schedule recommended by the National Immunisation Program, a variation of that schedule involving delay or splitting of vaccines, and finally, the possibility that you do not vaccinate your child. In each scenario you will be asked to choose which option you most prefer. The questions might seem a bit repetitive, but each scenario is different, so please answer each one after examining the differences between the schedules. As you consider each new scenario, please think of it independently of the scenarios you will have seen before; take each scenario on its own terms.

There are no right or wrong answers, we are just interested in your opinions. We have found most people do answer these questions honestly, so please just tell us what you believe is your most preferred option.

“>>” to Continue

Programmer: Select at random a BLOCK from the design to be presented to the respondent. Each BLOCK will have 12 tasks.

a. Record BLOCK in database.
b. Present 12 tasks in random order to respondent. Record in database …
   i. Order presented (e.g., 3-7-1-12-…)
   ii. Choice response in database in the original order of the design (response to 3^rd^ design row of BLOCK should be in 3^rd^ position, regardless of the order in which this design row was actually presented to respondent).

Scenario KKK of 12

Examine the three vaccination schedules shown below and indicate which one schedule you would most prefer to follow for the child you or your partner is currently carrying.

**Programmer**: Line below should only be present in scenarios 2-12.

Please ignore prior scenarios. Treat this scenario independently of previous scenarios.

| **Child’s Age** | **Schedule A**  **Recommended vaccination schedule** | **Schedule B**  **Delay or split some vaccinations or visits** | **Schedule C**  **No vaccinations** |
| --- | --- | --- | --- |
| **Birth** | Hepatitis B (1 injection) | **Delay [b.delay]** set of vaccines, and **split [b.split]** set vaccines over 12 months | No vaccinations are given |
| **2 months** | Hepatitis B, Diphtheria, Tetanus, pertussis, polio, Haemophilus Influenzae B; Pneumococcal conjugate; Rotavirus |  |  |
| **4 months** | Hepatitis B, Diphtheria, Tetanus, pertussis, polio, Haemophilus Influenzae B; Pneumococcal conjugate; Rotavirus |  |  |
| **6 months** | Hepatitis B, Diphtheria, Tetanus, pertussis, polio, Haemophilus Influenzae B; Pneumococcal conjugate; Rotavirus |  |  |
| **12 months** | Haemophilus Influenzae B, Meningococcal C, Measles, mumps, rubella (MMR) |  |  |
| **Consequences over 12 months** |  |  |  |
| Total number of injections | [**a.inj]** | [**b.inj]** injections than recommended schedule | [**c.inj]** |
| Total number of visits to clinic for vaccinations | **[a.vis]** | **[b.vis]** visits than recommended schedule | [**c.vis]** |
| Chance your child will get a vaccine preventable disease | **[a.risk]** in 1000 children | **[b.risk]** in 1000 children | **[c.risk]** in 1000 children |
| Chance your child might experience minor side effects (eg mild temperature, irritability, pain at injection site) | **[a.se]** | **[b.se]** | **[c.se]** |
| Implications for formal childcare arrangements as a result of vaccine status | **[a.care]** | **[b.care]** | **[c.care]** |
| Implications for government family assistance payments as a result of vaccine status | **[a.paym]** | **[b.paym]** | **[c.paym]** |
| **My preferred vaccination schedule for my child is (tick only one of these) …** | 🞎 | 🞎 | 🞎 |

“>>” to Continue

Programmer: Only show continue button (“>>”) once choice has been made.

Section 3 - Social networks

In this section we will ask you about different people in your life and the role they might play in your decision to vaccinate your child.

3.1 For each person or category listed in the left-hand column, please answer the questions in the other columns.

| Person | Do you have a …? | Would this person(s) be a possible source of health information? | Gender? | How long have you known this person? (drop down categories:  -Less than 1 year  -1 to less than 2 years  -2 to less than 3 years  -3 to less than 5 years  -5 or more years) | How important is this person’s view on your decision to vaccinate your child? * | | | | |
| --- | --- | --- | --- | --- | --- | --- | --- | --- | --- |
|  |  |  |  |  | Very important |  | Somewhat important |  | not at all important |
| Partner | 🔾 y 🔾 n | 🔾 y 🔾 n | 🔾 m 🔾 f |  |  |  |  |  |  |
| Mother | 🔾 y 🔾 n | 🔾 y 🔾 n |  |  |  |  |  |  |  |
| Father | 🔾 y 🔾 n | 🔾 y 🔾 n |  |  |  |  |  |  |  |
| Friend with children | 🔾 y 🔾 n | 🔾 y 🔾 n | 🔾 m 🔾 f |  |  |  |  |  |  |
| Friend without children | 🔾 y 🔾 n | 🔾 y 🔾 n | 🔾 m 🔾 f |  |  |  |  |  |  |
| Usual GP | 🔾 y 🔾 n | 🔾 y 🔾 n | 🔾 m 🔾 f |  |  |  |  |  |  |
| Usual other health care professional (like a community nurse) | 🔾 y 🔾 n | 🔾 y 🔾 n | 🔾 m 🔾 f |  |  |  |  |  |  |
| Usual pharmacist | 🔾 y 🔾 n | 🔾 y 🔾 n | 🔾 m 🔾 f |  |  |  |  |  |  |
| School teacher of your acquaintance | 🔾 y 🔾 n | 🔾 y 🔾 n | 🔾 m 🔾 f |  |  |  |  |  |  |
| Clergy (formal religious leader) or a respected person of your faith | 🔾 y 🔾 n | 🔾 y 🔾 n | 🔾 m 🔾 f |  |  |  |  |  |  |

Programmer: Randomize row order above.

“>>” to Continue

Programmer: For each person in the table in Q3.1 for which column 2 is Yes, ask Q3.2, one per screen.

3.2 For <person type-column 1 of 3.1>, please tell us how likely YOU believe THEY would be to recommend each vaccination option below by allocating 100 points across the options for this person, with more points meaning that they are more likely to recommend that option – the points should add up to 100 across the three options.

| Recommendation that <person type> would support … | Support level you believe they would have for this vaccination option |
| --- | --- |
| a. Follow government recommended schedule for your child | ____ |
| b. Vary the child’s vaccination schedule as you see fit (delay or split vaccines) | ____ |
| c. Don’t vaccinate your child at all | ____ |
| Total points (adds up to 100) |  |

Programmer: Ensure that Q3.2a+Q3.2b+Q3.2c=100. Show option to continue only after this condition is met.

“>>” to Continue

Section 4 – Sociodemographics

Now we have some further questions about you.

4.1 What is the highest year of primary or secondary school you have completed?

1. Year 12 or equivalent
2. Year 11 or equivalent
3. Year 10 or equivalent
4. Year 9 or equivalent
5. Year 8 or equivalent
6. Primary school only

4.2 What is the highest post-school qualification you have?

1. Postgraduate degree or equivalent
2. Graduate diploma and graduate certificate from university or equivalent
3. Bachelor degree or equivalent
4. Advanced diploma and diploma from university/TAFE or equivalent
5. Certificate or equivalent (e.g. Certificate III & IV or Certificate I & II)
6. None of the above, high school only

4.3 Which of the following best describes your household's gross annual income (before tax)?

- Nil Income (4)
- $1-$7,799 (i.e. $1-$149 a week) (5)
- $7,800-$12,999 (i.e. $150-$249 a week) (6)
- $13,000-$18,199 (i.e. $250-$349 a week) (7)
- $18,200-$25,999 (i.e. $350-$499 a week) (8)
- $26,000-$33,799 (i.e. $500-$649 a week) (9)
- $33,800-$41,599 (i.e. $650-$799 a week) (10)
- $41,600-$51,999 (i.e. $800-$999 a week) (11)
- $52,000-$62,399 (i.e. $1,000-$1,199 a week) (12)
- $62,400-$72,799 (i.e. $1,200-$1,399 a week) (13)
- $72,800-$88,399 (i.e. $1,400-$1,699 a week) (14)
- $88,400-$103,999 (i.e. $1,700-$1,999 a week) (15)
- $104,000-$129,999 (i.e. $2,000-$2,499 a week) (16)
- $130,000-$155,999 (i.e. $2,500-$2,999 a week) (17)
- $156,000-$181,999 (i.e. $3,000-$3,499 a week) (18)
- $182,000-$207,999 (i.e. $3,500-$3,999 a week) (19)
- $208,000 or more (i.e. $4,000 or more a week) (20)
- Prefer not to answer (21)

4.4 Postcode where you currently live: _______

4.5 How many children do you already have less than 18 years of age?

This is my first (0); 1, 2, 3, 4; 5+

Programmer: if Q4.5=0, go to CCC.

4.6 For each of your children already born, please indicate below their vaccination status:

Programmer: Present only as many rows as indicated from Q4.5.

| Child  (oldest first) | Child’s Age | Vaccination Status  (Drop down menu:  -Completely up to date  -Mainly up to date  -Partially vaccinated  -Not vaccinated  -Don’t know) |
| --- | --- | --- |
| 1 | _____ |  |
| 2 |  |  |
| 3 |  |  |
| 4 |  |  |
| 5 |  |  |

CCC:

4.7 In what country were you born? (dropdown menu)

4.8 In what country were your parents born? (dropdown menu)

4.9 Were you vaccinated when you were a child? Y/ N/ don’t know

Section 5 – Attitudes and Perceptions

5.1 Consider the statements below one at a time and indicate whether you think each is true or false.

|  | True | False | Don’t know |
| --- | --- | --- | --- |
| Vaccines are superfluous as diseases can be treated (eg with antibiotics) |  |  |  |
| Without broadly applied vaccine programs, smallpox would still exist |  |  |  |
| The efficacy of vaccines has been proven |  |  |  |
| Children would be more resistant if they were not always vaccinated against all diseases |  |  |  |
| Diseases like autism, multiple sclerosis and diabetes might be triggered through vaccinations |  |  |  |
| The immune system is not overloaded through many vaccinations |  |  |  |
| Many vaccinations are administered too early, so that the body’s own immune system has no chance of developing |  |  |  |
| The doses of the chemical used in vaccines are not dangerous for humans |  |  |  |
| Vaccinations increase the occurrence of allergies |  |  |  |

Programmer: randomize order of statements above for each respondent

5.2 How much do you agree with the each of the following statement on vaccinations?

|  | strongly disagree | disagree | neither agree nor disagree | agree | strongly agree |  |
| --- | --- | --- | --- | --- | --- | --- |
| Childhood vaccines are important for my child's health | 🔾 | 🔾 | 🔾 | 🔾 | 🔾 |  |
| Childhood vaccines are effective | 🔾 | 🔾 | 🔾 | 🔾 | 🔾 |  |
| Having my child vaccinated is important for the health of others in my community | 🔾 | 🔾 | 🔾 | 🔾 | 🔾 |  |
| All childhood vaccines offered by the government programme in my community are beneficial. | 🔾 | 🔾 | 🔾 | 🔾 | 🔾 |  |
| New vaccines carry more risks than older vaccines | 🔾 | 🔾 | 🔾 | 🔾 | 🔾 |  |
| The information I receive about vaccines from the vaccine program is reliable and trustworthy. | 🔾 | 🔾 | 🔾 | 🔾 | 🔾 |  |
| Getting vaccines is a good way to protect my child/children from disease. | 🔾 | 🔾 | 🔾 | 🔾 | 🔾 |  |
| Generally I do what my doctor or health care provider recommends about vaccines for my child/children. | 🔾 | 🔾 | 🔾 | 🔾 | 🔾 |  |
| I am concerned about serious adverse effects of vaccines. | 🔾 | 🔾 | 🔾 | 🔾 | 🔾 |  |
| My child/children does or do not need vaccines for diseases that are not common anymore. | 🔾 | 🔾 | 🔾 | 🔾 | 🔾 |  |

Programmer: randomize order of statements above for each respondent

5.3 How much do you agree with the each of the following statements?

|  | strongly disagree | disagree | neither agree nor disagree | agree | strongly agree |  |
| --- | --- | --- | --- | --- | --- | --- |
| It is unfair that parents have to vaccinate their children to get the childcare rebate | 🔾 | 🔾 | 🔾 | 🔾 | 🔾 |  |
| Established medical knowledge can be wrong | 🔾 | 🔾 | 🔾 | 🔾 | 🔾 |  |
| We are given accurate information about the chances of negative side effects after vaccinations | 🔾 | 🔾 | 🔾 | 🔾 | 🔾 |  |
| My spiritual beliefs do not support vaccination | 🔾 | 🔾 | 🔾 | 🔾 | 🔾 |  |
| Hearing stories about children who have gotten very sick with a vaccine preventable disease makes me automatically think about that happening to my own child | 🔾 | 🔾 | 🔾 | 🔾 | 🔾 |  |
| The health and wellbeing of other people’s children is not my responsibility | 🔾 | 🔾 | 🔾 | 🔾 | 🔾 |  |
| I don’t like being told by other people that I need to vaccinate my child | 🔾 | 🔾 | 🔾 | 🔾 | 🔾 |  |
| The way the government advises parents to vaccinate their children is reasonable | 🔾 | 🔾 | 🔾 | 🔾 | 🔾 |  |
| Vaccines are not as effective as they are made out to be | 🔾 | 🔾 | 🔾 | 🔾 | 🔾 |  |
| People should vaccinate their children because it is for the common good | 🔾 | 🔾 | 🔾 | 🔾 | 🔾 |  |
| The pressure to vaccinate my child restricts my free choice |  |  |  |  |  |  |
| Giving multiple vaccines at once will increase their negative side effects |  |  |  |  |  |  |
| It is reasonable for the medical community to recommend vaccines |  |  |  |  |  |  |

Programmer: randomize order of statements above for each respondent

5.4 How much do you agree with the each of the following statements?

|  | strongly disagree | disagree | neither agree nor disagree | agree | strongly agree |  |
| --- | --- | --- | --- | --- | --- | --- |
| Doctors and pharmaceutical companies recommend vaccines so they can profit by treating any side effects children might have |  |  |  |  |  |  |
| It is more important to listen to scientific research on vaccines than my own feelings |  |  |  |  |  |  |
| Vaccines contain toxins and chemicals that can harm my child |  |  |  |  |  |  |
| As a member of the community I have a responsibility to vaccinate my child to protect those who can’t be immunised |  |  |  |  |  |  |
| I would never choose alternative therapies for my child over a recommended medical treatment like vaccines |  |  |  |  |  |  |
| Vaccinating infants and young children is not ethical |  |  |  |  |  |  |
| I should not be told what to do with my own child regarding vaccinations |  |  |  |  |  |  |
| I know enough to make my own decision about vaccination without listening to doctors or the government |  |  |  |  |  |  |
| If I did not vaccinate my child I’d be worried about them catching a vaccine preventable disease |  |  |  |  |  |  |
| There are healthier and more natural alternatives to vaccination such as naturopathy or homeopathy |  |  |  |  |  |  |
| I trust my nurse or doctor to give me good advice about vaccinations |  |  |  |  |  |  |
| Good doctors should tell parents to vaccinate their children |  |  |  |  |  |  |
| The dangers of diseases are exaggerated to frighten parents |  |  |  |  |  |  |
| Religious or spiritual beliefs should not get in the way of vaccinating your child |  |  |  |  |  |  |
| Diseases like whooping cough and polio can be life threatening |  |  |  |  |  |  |
| There is strong social pressure to vaccinate my child |  |  |  |  |  |  |
| Vaccines do not work |  |  |  |  |  |  |
| Doctors and pharmaceutical companies have ulterior motives |  |  |  |  |  |  |

Programmer: randomize order of statements above for each respondent

5.5 In this question, we will refer repeatedly to “health behaviours”, by which we mean anything that you do that impacts your health, like going to the doctor, your eating habits, or physical activity.

For each statement below, please indicate the degree to which you agree or disagree with each statement.

I rarely engage in health behaviours until I am sure my friends approve of them.

It's important that others agree with my health lifestyle [before I act].

When engaging in health behaviours, I generally do things that I think others will approve of.

I like to know what health behaviours make good impressions on others.

I achieve a sense of belonging by making the same healthy choices that others do.

If I want to be like someone. I often try to make the same healthy choices that they do.

I often identify with other people by making the same healthy choices that they do.

If I have little experience with a health behaviour, I often ask my friends about it.

I often consult other people to help choose the best alternative available for a health behaviour.

I frequently gather information from friends and family before I engage in a health behaviour.

Programmer: scale is the same as Q5.4.

Programmer: randomize order of statements above for each respondent

5.6 When you were thinking about the future vaccination of your unborn child in Section 2 of the sruvey, which of the reasons below were motivating you? (check all that apply)

| I was motivated to … | Check all that apply |
| --- | --- |
| Avoid putting my child at risk of these infections |  |
| Avoid putting other people’s children at risk from these infections |  |
| Help keep other people in society safe from these infections |  |
| Make sure the vaccination rate in Australia is high enough for herd immunity |  |
| Avoid exposing my child to risks of possible adverse effects from vaccines |  |
| Avoid government intervention in my life |  |
| Comply with the desires of my partner |  |
| Behave like people similar to me |  |
| Maintain eligibility for family assistance payments |  |
| Obey the rules or indications of my religion |  |
| Make a decision that’s socially responsible |  |
| Make a decision that’s socially acceptable |  |
| Comply with the opinions of important people in my life (other than your partner) |  |
| Avoid other people telling me what I should do |  |
| Other  (please provide): ________________ |  |

Programmer: randomize order of statements above for each respondent (except that Other should always be presented last).

5.7 Other people like you might decide to delay or not to vaccinate their child during its first year of life. Below are some reasons that might be given for not vaccinating at all. Please indicate whether any of these reasons might apply to you.

| Reason given for not vaccinating their child … | Does this reason apply to you? |
| --- | --- |
| Religious objection | Y/N |
| Fear of needles | Y/N |
| No access to medical services | Y/N |
| No transportation to medical services | Y/N |
| No childcare services for other children | Y/N |
| Lack of spousal/ partner cooperation | Y/N |
| Too expensive for your budget | Y/N |
| No time to access medical services | Y/N |
| No family support | Y/N |
| Other (provide): _____________________ |  |

Programmer: randomize order of statements above for each respondent, but maintain Other always last.

5.8 Prior to taking this survey, had you ever seen or read a brochure about the National Immunisation Program for children up to 12 months of age, issued by the Australian government?

🞎 Yes 🞎 No

That’s it! We greatly appreciate your participation in this survey. If you have any comments about the survey or its subject matter, please use the text box below to tell us.

Thank you again!

Text box for user comments.
